# Development of a Fault Monitoring Technique for Wind Turbines Using a Hidden Markov Model

**DOI:** 10.3390/s18061790

**Published:** 2018-06-02

**Authors:** Sung-Hwan Shin, SangRyul Kim, Yun-Ho Seo

**Affiliations:** 1 Department of Automotive Engineering, Kookmin University, 77 Jeongneung-ro, Seongbuk-gu, Seoul 02707, Korea; 2 System Dynamics Lab, Korea Institute of Machinery & Materials, 156 Gajeongbuk-ro, Yuseong-gu, Daejeon 34103, Korea; srkim@kimm.re.kr (S.K.); yhseo@kimm.re.kr (Y.-H.S.)

**Keywords:** wind turbine, fault monitoring, vibration signal, Weibull distribution, hidden Markov model, signal processing

## Abstract

Regular inspection for the maintenance of the wind turbines is difficult because of their remote locations. For this reason, condition monitoring systems (CMSs) are typically installed to monitor their health condition. The purpose of this study is to propose a fault detection algorithm for the mechanical parts of the wind turbine. To this end, long-term vibration data were collected over two years by a CMS installed on a 3 MW wind turbine. The vibration distribution at a specific rotating speed of main shaft is approximated by the Weibull distribution and its cumulative distribution function is utilized for determining the threshold levels that indicate impending failure of mechanical parts. A Hidden Markov model (HMM) is employed to propose the statistical fault detection algorithm in the time domain and the method whereby the input sequence for HMM is extracted is also introduced by considering the threshold levels and the correlation between the signals. Finally, it was demonstrated that the proposed HMM algorithm achieved a greater than 95% detection success rate by using the long-term signals.

## 1. Introduction

Wind turbines are one of the most remarkable renewable energy generation systems and many studies have been being conducted to reduce their operating cost. A survey on the total cost of wind power generation cost concluded that the operating cost, which includes maintenance (O&M), training operators and engineers, repair, system upgrades, inventory etc., is larger than capital costs, such as facility design, development planning, and construction. Concretely, the maintenance cost accounts for more than 25% of the total cost [1,2].

In practice it is not easy to perform regular inspections for the maintenance of wind turbines that are located at inaccessible places, such as mountaintops, shorelines, or oceans, and deserts. In addition, unexpected faults may happen due to an abrupt change in the environmental conditions including extreme weather events as well as performance degradation. These issues have been addressed by using condition monitoring systems (CMSs). This are a part of predictive maintenance (PdM) [3,4] and are installed to detect abnormal conditions of the mechanical parts of wind turbines in advance. PdM, which is also known as condition-based maintenance (CBM), attempts to evaluate the condition of an asset by performing periodic or continuous monitoring [1]. According to a report by the Electrical Power Research Institute (EPRI) [5], when a power plant is managed through PdM, its maintenance costs have a minimum five-fold benefit in comparison with the maintenance costs through visual inspection and ten-fold in comparison with the total cost after problems occur. For this reason, it is expected that the economics of wind power generation can be improved if a CMS allows early fault detection of mechanical systems. 

A CMS consists of sensors, data acquisition equipment, and signal processing algorithms. Sensors are typically used for vibration analysis, temperature analysis using thermocouples and/or thermography, ultrasonic testing, oil analysis, strain measurement, acoustic emission test, electrical effect measurements, and so on [6,7,8,9,10]. Among them, vibration is the most effective quantity measured in rotary wind turbines [10]. 

Signal processing algorithms are utilized to examine the health conditions of the mechanical components of wind turbines by extracting important information from the various types of sensors which collect information in two representative dimensions: time and frequency. Time-domain signal processing such as statistical methods, trend analysis, and filtering makes use of overall and/or average quantities and is applied to monitor the health condition of wind turbines. Plots of data, such as rotational speed with time, allow for comparison with pre-defined thresholds: warning and alarm levels. The time-domain signal processing method is very useful for determining whether a wind turbine has crossed a threshold that indicates a dangerous condition. 

Frequency-domain signal processing based on the fast Fourier transform (FFT) is employed to diagnose where and how a wind turbine behaves abnormally. Considering frequency-based methods such as spectrum, envelope, and Cepstrum analyses using vibration signals, requires previous understanding of how the frequency is affected when a component behaves abnormally. In the case of variable rotational speed, waterfall analysis using wavelet, short time Fourier transform (STFT) and order analysis [11] can be applied for detecting abnormal symptoms of wind turbines. These kinds of conventional methods have two main problems. One deals with each sensor’s physical data individually, but does not consider interrelationships with other types of sensors data. The other is a difficulty in analyzing non-linear and non-stationary data, which reflect changes of wind speed and rotation speed. For these reasons, more advanced signal processing techniques such as angular resampling [12] have been being investigated for monitoring the health condition of wind turbines with greater reliability. 

There are numerous expert approaches for the machine diagnosis and prognosis when implementing CBM and they can also apply to fault monitoring of the wind turbines. The ways can be divided into three categories: physical models, artificial intelligence (AI) models, and hybrid models. The physical model is based on mathematical or logical representations of the mechanical system and therefore it is also named a white-box method. On the contrary, AI models are considered black-box methods because they can be applied to diagnose or predict without knowing the internal relationship of the mechanical system. Among the AI models, the artificial neural network (ANN) has been mainly applied to classification and recognition problems because it has a good theoretical background and can perform non-linear feature mapping with high accuracy [13,14]. The Hidden Markov model (HMM) is widely used for pattern and/or speech recognition because it has an excellent mathematical basis and is an efficient modeling tool for sequences with temporal constraints [15]. The hybrid model integrates two or more individual white-box and/or black-box methods for eliminating the limitations inherent in each method. However, it is still difficult to indicate a specific method which can generally apply to any problem because its performance depends on database representing the problem [13]. 

The purpose of this study is to propose a fault detection algorithm for the mechanical system of wind turbines in order to upgrade the function of CMS. To this end, wind turbines with a capacity of 3 MW are employed and they consist of a rotor blade, main shaft and bearing, gearbox, and permanent magnetic synchronous generator (PMSG). Long-term data over two years including vibration, wind speed, rotating speed, etc. were collected by their CMS. First, the structure of a wind turbine, its operating phases, and installed CMS are briefly explained in Section 2. Subsequently relationships among the operating conditions of wind turbine, wind speed, shaft rotating speed, and power are investigated. Trend analysis on vibration signals is conducted as a function of the number of the revolutions of the main shaft in order to find out its distribution and alarm thresholds are determined which indicate abnormalities of the mechanical system in Section 3. Third, correlation analysis of the vibration signals is used to find coupling of physical components. Then, a HMM is employed to propose the statistical fault detection algorithm in the time domain and take its input sequence considering the correlation between vibration signals in Section 4.1. Lastly, the performance of the proposed HMM is investigated by introducing in Section 4.2 some metrics that can account for the imbalance of datasets. 

## 2. Wind Turbine and Its Condition Monitoring System

### 2.1. Operation of Wind Turbine

The mechanical system of a wind turbine capable of generating power consists of a rotor blade, main shaft, gearbox, and generator. The rotor blade and main shaft rotate at low speed and the gearbox increases the speed so that generator makes power. The wind turbine is controlled to ensure safe operation and stable power generation in several phases [16,17]. An *initialization* phase is used to check each part of the wind turbine before operation and determine the positions of rotor and yaw. A *preparation* phase monitors the proper operation condition by checking the wind direction and speed and starts to monitor the connection between transmission parts. A *checking* phase judges whether operation is possible considering the variations and duration of the wind. These three phases occur prior to operation. 

A *start-up* phase releases the brake system after confirming the wind speed and starts to run the wind turbine. A *generating* phase generates power when the operating speed is normal. During the power generation in these two phases, that is, the wind turbine is connected to the power grid, the generator is subjected to a certain load. A *free-running* phase allows free rotation of the rotor when the wind speed is not enough to generate power and at that time, yaw controls the direction of the rotor through the wind direction. Finally, a *stopping* phase stops the rotor by adjusting the aerodynamic drag device and the pitch of rotor blade. These four steps are for the operation of the wind turbine and for the power generation. 

### 2.2. Condition Minitoring System of Wind Trubine

**Figure 1:**
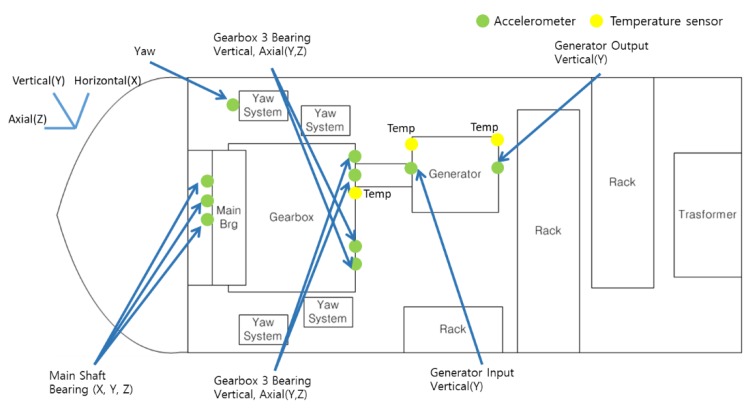
Simple structure of wind turbine with a permanent magnetic synchronous generator (PMSG) and the positions of installed accelerometers and temperature sensors.

CMS is utilized for solving the difficulties related to the maintenance of wind turbines. Figure 1 shows the mechanical parts of the wind turbine used in this study and the positions of sensors for its CMS. Accelerometers and temperature sensors are installed to measure the vibration signals and temperature, respectively, of the following mechanical parts: rotor, main shaft bearing, gearbox, and generator. Environmental and operation variables such as wind direction, wind speed and generated power are also measured. 

These data are obtained with a sampling frequency of several thousand kHz and more using data acquisition equipment, but averaged data is used and stored at the interval of 1 s to monitor the status of the wind turbine. For vibration signals, the acceleration measured in each mechanical part is treated as two values based on the criteria of IEC 61400-25-6 [18,19]. One is the root mean square (RMS) value of acceleration components below the frequency 1 kHz and the other is a RMS value after applying a high pass filter with a cut-off frequency 1 kHz. The latter is also called as high frequency bandpass components (HFBP). Table 1 summarizes the main vibration signals treated in this study and here, X, Y, and Z indicate the vibration directions: horizontal, vertical, and axial to the main shaft. Wind speed and generated power are also averaged at an interval of 1 s. The data used in this study is gathered during a period greater than two years; the number of data per channel is about 45 million. 

## 3. Vibration Signals and Their Alarm Thresholds

### 3.1. Relation between Wind Speed, Rotating Speed of Main Shaft, and Generated Power

#### 3.1.1. Distribution of Wind Speed

The most important environmental variable in wind power generation is a wind speed. As stated in Section 2.1, the wind speed and its duration are key parameters that affect the operation phase of a wind turbine and the generated power. Therefore, the distribution of wind speed at the site where the wind turbines will are located should be investigated in advance. 

**Figure 2:**
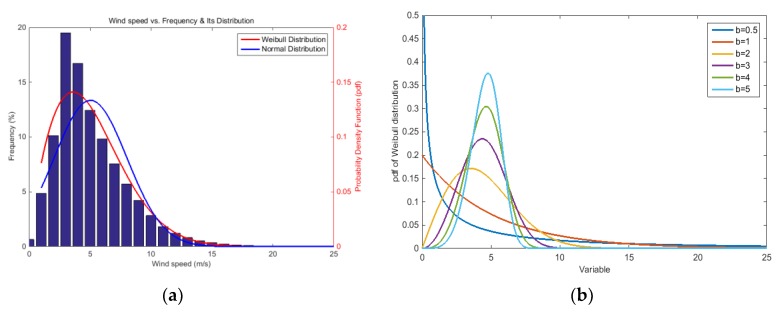
(**a**) Distribution of wind speed obtained from wind turbine No.1 during about 2 years and statistical distribution functions for simulating its shape; (**b**) Probability density functions of Weibull distribution according to shape factor (*b*) at the scale factor (*a*) of 5.0.

Figure 2a shows the distribution of wind speed measured at wind turbine No.1 and *Frequency* is the ratio of the frequency at interval of 1 m/s defined as follows:

(1) Frequency(%)=(No. of data at each wind speed)/(No. of all data)×100.

During the measurement period, maximum *frequency* (mode) of wind speeds occurs at 3 m/s and there are few wind speeds faster than 18 m/s. If one applies the normal distribution function commonly used for approximating this kind of distribution, the mean value (*m*) is 5.0 m/s and standard deviation (*σ*) is 3.0 m/s. This difference between mode and the mean values of the wind speed can be explained due to the discrepancy between the normal distribution’s symmetry and the skewness of the actual wind distribution; see the comparison of the probability distribution function in Figure 2a. 

To solve the problem, the Weibull distribution is introduced that is used for simulating reliability data. It is usually applied to residual time prediction, weather forecasting, failure analysis, etc. and defined as follows [20]:

(2) $$f\left(x|a,b\right)=\frac{b}{a}{\left(\frac{x}{a}\right)}^{b-1}e^{-{\left(\frac{x}{a}\right)}^{b}},$$

where *x* is a positive real number, *a* a scale factor and *b* a shape factor. Figure 2b shows examples of the Weibull distribution as a function of the shape factor using scale factor of 5. It shows that the Weibull distribution is asymmetric at low shape factors and approaches the normal distribution at greater shape factors. The probability density function of Weibull distribution with a scale factor of 5.7 and a shape factor of 1.8 is plotted and compared with the distribution of the wind speed in Figure 2a. From the result, it is found that the Weibull distribution can be utilized as a tool to simulate the distribution of wind speed.

#### 3.1.2. Wind Speed vs. Rotating Speed of Main Shaft

**Figure 3:**
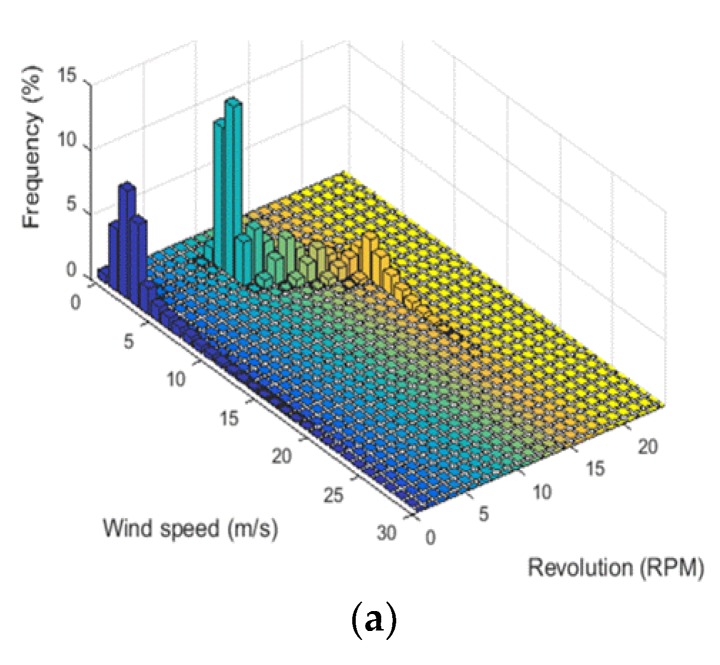

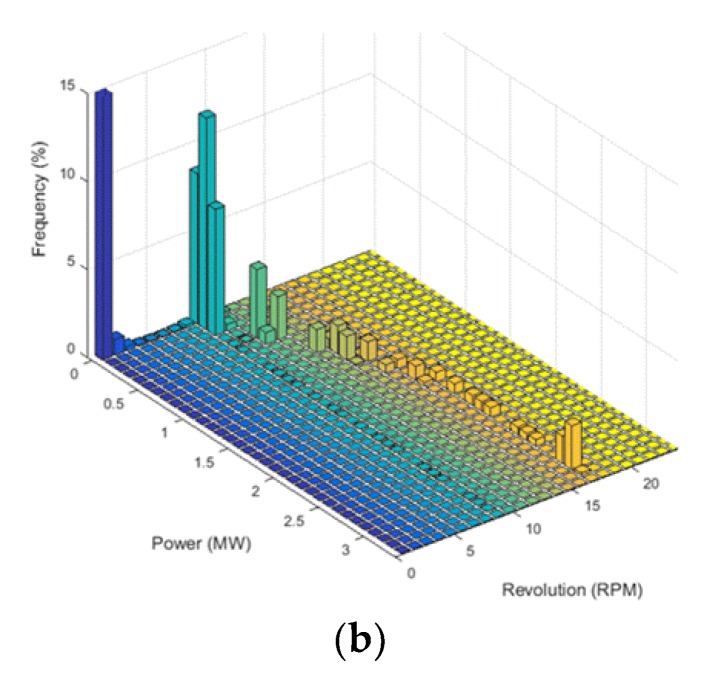
(**a**) Data distribution as a function of wind speed and rotating speed of main shaft; (**b**) Data distribution as a function of rotating speed of main shaft and generated power.

As stated at Section 2.1, the probability that the main shaft rotates is increased when the wind speed is fast and its duration is long. Figure 3a shows the relation between wind speed and rotating speed of main shaft. The main shaft may or may not rotate if the wind speed is 3 m/s, the highest *frequency* of wind speed. If the rotational speed of the main shaft is zero, the operating phases are *initialization*, *preparation*, and *checking*; in the case of 8 revolution per minute (rpm), the phases are *start-up*, *generation*, and *free-run*. That is, it means that the operating conditions may differ even at identical wind speeds. In the range of wind speeds of 5~8 m/s, the number of revolutions increases according to the wind speed. However, at wind speeds of 9 m/s and more, it remained at 16 rpm. This is because the control system precludes excessive rotation for safe operation.

#### 3.1.3. Rotating Speed of Main Shaft vs. Generated Power

Figure 3b shows the relation between rotating speed of main shaft and generated power. Power generation is started at *start-up* and is done mainly during *generation*. In fact, the amount of generated power is very low when the revolution is lower than 8 rpm. However, there is a considerable amount of time in which there is no generated power even if the rotational speed is higher than 8 rpm. This is because the generator does not work during *start-up* and *free-running*. When the rotational speed increases from 10 to 15 rpm, the amount of generated power is more than 0.5 MW and maximum power is generated at a rotational speed of 16 rpm. This means that all power transmission systems of wind turbine combine and operate when the rotational speed is above 10 rpm. 

Compared with Figure 3a, it can be seen that the rotation of the main shaft depends on the wind speed and it influences the generated power. Stable power generation is possible if the wind speed remains at 5 m/s or more. From these results, the number of revolutions of the main shaft could be utilized as a useful parameter when analyzing vibration signals obtained from CMS in this study. 

### 3.2. Trend Analysis of Vibration Signals

#### Distribution of Vibration Signals

**Figure 4:**
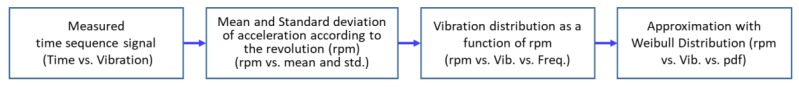
Procedure of trend analysis on vibration signal; pdf = probability density function.

The vibration signal is the most important physical quantity that reflects health condition of a wind turbine’s power transmission systems such as main shaft, gearbox, and generator. In this section, the variation of vibration is investigated and approximated in the statistical manner as shown in Figure 4.

**Figure 5:**
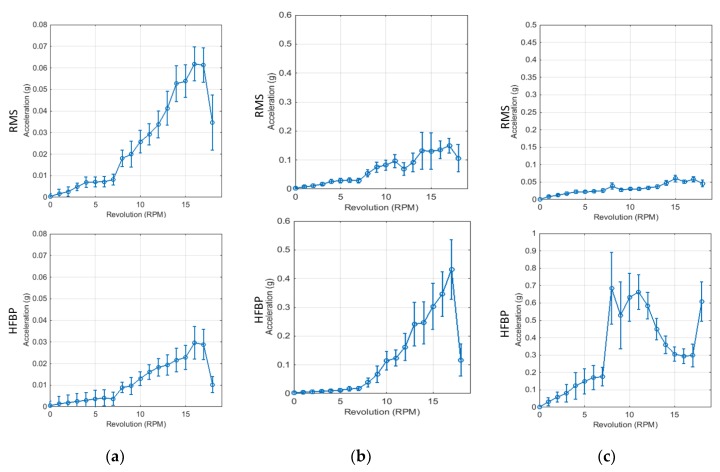
Mean (dot) and standard deviation (error bar) of vibration at (**a**) the bearing of main shaft, (**b**) the high-speed part of gearbox, and (**c**) generator as a function of rpm. All vibration signals are measured in the y-direction.

First, measured vibration signals in the time domain are transformed as a function of the number of revolutions of the main shaft in rpm and then the mean and standard deviation of the data at each rpm are calculated. Figure 5a shows the variation trends of mean values of acceleration in the y-direction measured on the bearing of the main shaft. The acceleration is small until 7 rpm and there is almost no change even if rpm increases. However, since the mechanical system including the generator starts to be subjected to a load for generating power at the range over 8 rpm, the acceleration gradually increases. At high wind speed, the number of revolution remains constant through blade pitch control. For the wind turbine used in this study, the revolution is 18 rpm. In this process, the amount of load varies to increase the amount of generating power and that is the reason why the vibration is changed at 18 rpm.

Figure 5b shows the variation of vibration measured at the high-speed part of gearbox. It has the same trend as the vibration of the bearing of main shaft. The difference is that HFBP is greater than RMS in the gearbox but reversed in the bearing. That is because the rotation speed of the gearbox is accelerated as fast as the gear ratio compared with the main shaft and therefore, the high frequency vibration components mainly occur. 

Figure 5c shows the variation of vibration measured at the generator as a function of the rotational speed of the main shaft. The generator connected with the high-speed part of gearbox also rotates with the high speed and so HFBP is as large as 10 times the RMS. In particular, unlike the tendency of main shaft and gearbox, HFBP increases abruptly at 8 rpm and tends to be similar or decrease at the range higher 8 rpm. This is because, as mentioned above, generator is subjected to a load to generate power.

**Figure 6:**
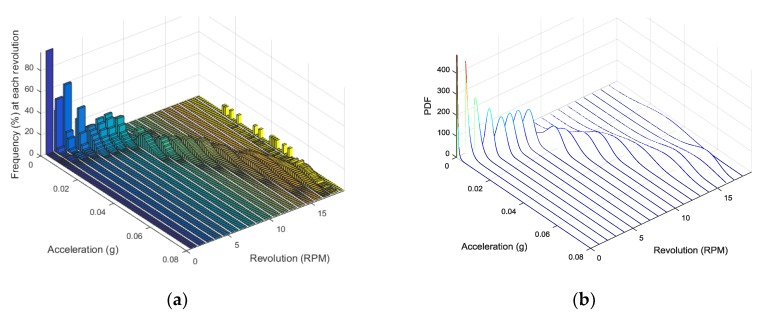
(**a**) Frequency distribution of RMS in y-direction of the bearing of main shaft as functions of the rpm and acceleration and (**b**) its approximation employing Weibull distribution.

Next, the *frequency* distribution of the vibration signal is investigated as a function of the rotational speed and acceleration of the main shaft. As an example, Figure 6a shows the vibration in the y-direction of the bearing of main shaft. At a specific rpm, the *frequency* increases up to a maximum value and then decreases exponentially. This tendency also occurs in the gearbox and generator, which is similar to the relationship between rpm and wind speed. 

Based on this result, the final step is to approximate the *frequency* variation of the vibration signal at each rpm with the Weibull distribution. Figure 6b is an example simulating the distribution of RMS value from Figure 6a. This approximation employs a statistical approach to the vibration distribution at a specific rpm of the main shaft.

### 3.3. Threshold Setting based on Alarm Level

CMS of the wind turbine requires vibration thresholds at each measuring point to judge the abnormality of mechanical systems such as main shaft, gearbox, and generator. In the previous studies [17,21] and in Section 3.1 of this study, it was found that the distribution of vibration can be approximated by Weibull distribution although this varies with the mechanical system.

Because the fault of a mechanical system can occur when the vibration signal is above a certain value, it seems to be statistically reasonable to apply the criterion based on a one-sided confidence interval of the probability distribution. If the data is normally distributed with mean, *m* and standard deviation, *σ*, the value of the cumulative distribution function (*cdf*) up to *m + σ* is 84.1%; up to *m +* 2*σ,* 97.7%; up to *m +* 3*σ*, 99.8%. This classification is based on the three sigma rule of the normal distribution. In this study, the vibration values corresponding to the three values of the *cdf* of the Weibull distribution are used as thresholds for indicating the alarm level as shown in Table 2. For reference, the *cdf* of the Weibull distribution is defined as follows:

(3) $$cdf\left(x|a,b\right)=1-e^{-{\left(\frac{x}{a}\right)}^{b}},$$

where *a* is a scale factor and *b* a shape factor.

**Figure 7:**
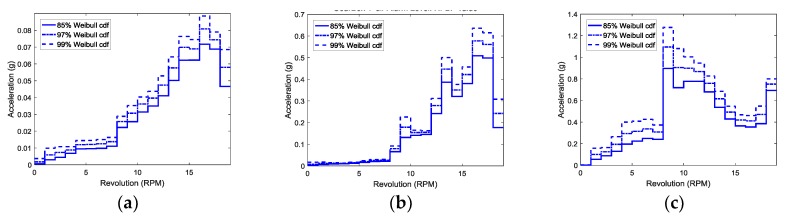
Vibration thresholds as a function of rpm for (**a**) the bearing of main shaft; (**b**) the high-speed part of gearbox, and (**c**) generator as a function of rpm. All vibration signals are measured in the y-direction.

Figure 7 shows the vibration thresholds as a function of rpm obtained from the *cdf* of the Weibull distribution for various mechanical parts. In Figure 7b, the thresholds at 11 rpm are lower than those at 10 rpm. Due to the nature of mechanical system, if the system is safe from vibration at 10 rpm, there is no problem in the safety of the system even if the same vibration occurs at 11 rpm. To reflect this phenomenon, the thresholds are modified as applying the following condition:

(4) $$If\;Tr_{@high-rpm}\leq Tr_{@low-rpm},\;then\;Tr_{@high-rpm}=Tr_{@low-rpm}.$$

**Figure 8:**
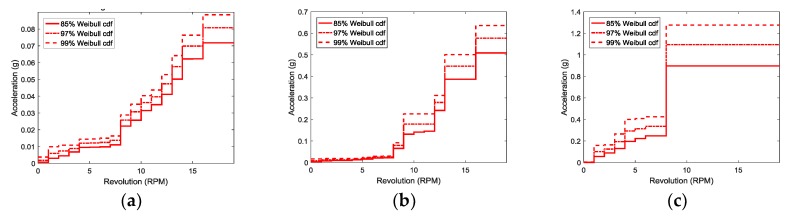
Modified vibration thresholds as a function of rpm for (**a**) the bearing of main shaft; (**b**) the high-speed part of gearbox, and (**c**) generator as a function of rpm. All vibration signals are measured in the y-direction.

Here, *Tr* is the threshold value at a specific rpm. Figure 8 shows the results modified from Figure 7.

## 4. Fault Monitoring Using Hidden Markov Model

### 4.1. Fault Detection Algorithm

CMS of the existing wind turbine produces an alarm signal when the vibration obtained through a specific channel is higher than a predetermined threshold. However, this method has the disadvantage that one-off external impact signals and alarm errors may occur: (a) when the system starts or stops operating or (b) due to unexpected electrical noise. In fact, because abnormal symptoms occurring in mechanical system induces continuously increasing vibration, it is desirable to generate an alarm signal considering the excessive vibration and its duration simultaneously. To this end, a fault monitoring algorithm is proposed using HMM, one of the stochastic models.

#### 4.1.1. Hidden Markov Model (HMM)

HMM has been widely used for the pattern classification or recognition such as speech, motion, and genes because of its excellent mathematical basis, high computational efficiency, and efficient modeling tool for a sequence with temporal constraints [15,22]. Recently, it has also been applied to engineering problems like fault recognition of mechanical systems. HMM has the distinct advantage of easy extension to the types of events to be classified or the types of recognizable faults. If the target signal is a one-dimensional sequence such as vibration and acoustic signal, its recognition performance is further improved [23]. 

**Figure 9:**
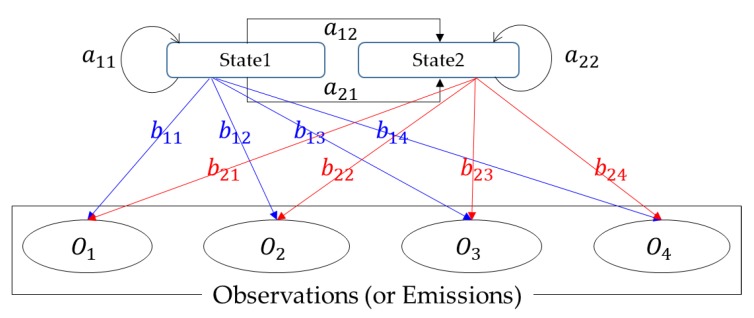
Example of HMM having two-state fully connected structure. *a_ij_* is the state transition probability and *b_pq_* is the observation symbol probability.

HMM is characterized by four model parameters: the number of states, the number of distinct observation symbols per state, state transition probability distribution *T* $=\left[a_{ij}\right]$, and observation symbol probability distribution *E* $=\left[b_{pq}\right]$ [15], as shown in Figure 9. There are three basic problems of interest that should be solved for the model to be useful in real-world applications. One is the evaluation problem that is to quantify how well a given model matches a given observation sequence representing a specific event. Another is the decoding problem, which is the uncovering of the hidden part of the model; in other words, the goal is to find the state sequence related to the series of events. The third issue is a learning problem that optimizes the model parameters of HMM by training with an ensemble of observation sequences related to the specific event. 

#### 4.1.2. Design of HMMs for Fault Detection

##### Structure and Input data

The states of HMM can be divided as follows: *normal* and *fault* because the operating conditions of the wind turbine can be normal or not. If one assumes that the same results can be observed even if their occurrence probabilities at each state are different from one another, a HMM having a two-state fully connected structure shown in Figure 9 can be utilized for this study. 

**Figure 10:**
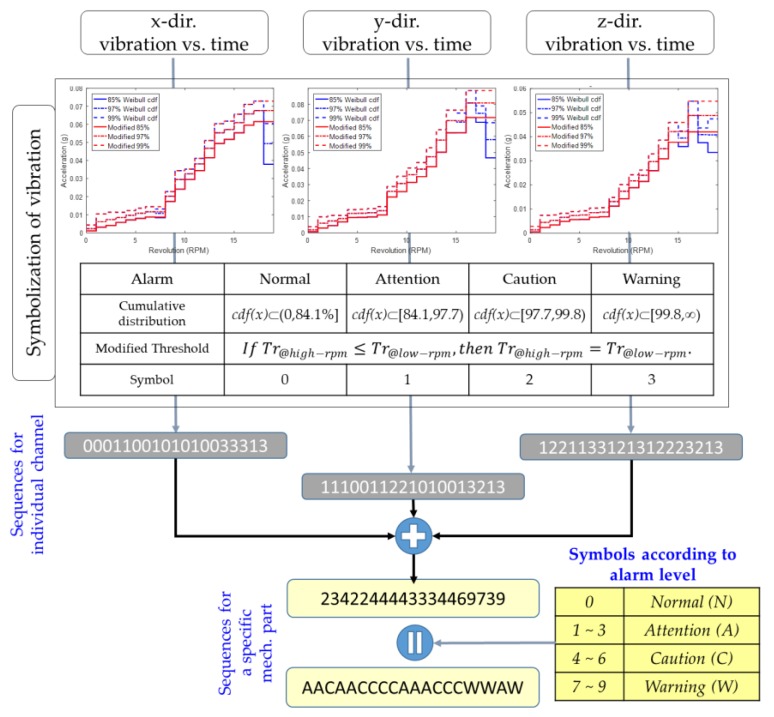
Method of extracting observation sequence of HMM to monitor a fault of main bearing of the wind turbine.

An observation sequence for the training of HMM is obtained through the process shown in Figure 10; this is an example of the bearing of the main shaft. First, vibrations at a specific time are compared with the thresholds as a function of rpm defined in Section 3.2 and then the operation at the moment is divided into 4 alarm levels: normal, attention, caution, and warning which are assigned the numbers: ‘0’, ‘1’, ‘2’, and ‘3’, respectively. With this method, vibration signals of individual channels are converted to the form of a number sequence indicating alarm level. 

Next, all channels used for measuring the vibrations of the specific mechanical part are simultaneously considered to prevent a false alarm that occurs due to unexpected electric noise on a particular channel [24]. In fact, the vibrations correlate with each other, even if they are measured in different directions. For the bearing of main shaft, the correlation between RMS values of vibration in the x-, y-, z-direction is high. For the gearbox, the correlation between HFBP values in y- and z-directions of low and high speed parts is also high. For the generator, the correlation between HFBP values in y- and z-direction of input and output parts is high [17]. 

To this end, the number sequence of individual channel are added according to the time. Because three channels are applied in the case of the bearing of the main shaft, the maximum and minimum numbers are 9 and 0, respectively. Here, ‘9’ means that at that time, all vibrations in the x-, y-, z-directions are on warning level and ‘0’ does that they are normal. 

Finally, the assignments of the symbols indicating the operating state at that time as introducing four indicators are as follows: ‘N‘ if the added number is 0, ‘A’ between 1 and 3, ‘C’ between 4 and 6, and ‘W’ between 7 and 9. The indicators are used as the observations of HMM. 

##### Process for Detecting Faults

**Figure 11:**
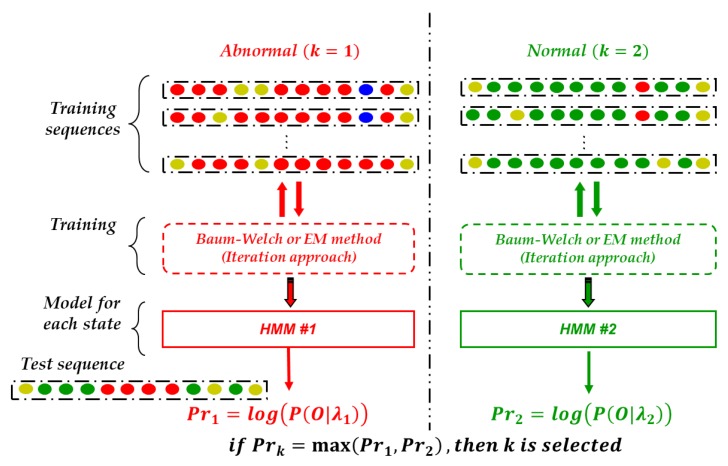
Process for detecting fault using HMM.

For fault detection of a mechanical part, two HMMs are needed. One is a model coincident with an abnormal state and the other is with a normal state as shown in Figure 11. The number of training sequences for the HMMs according to the mechanical part is summarized in Table 3. In particular, the intervals including more than twenty ‘W’ indicators during 100 s are extracted as the training sequence for the abnormal state. A HMM is trained by employing the Baum-Welch algorithm [25]; this is one of the iteration approaches. 

As a result, two HMMs for a mechanical part are obtained and Table 4 shows examples of the transient probability distribution and observation symbol distribution of the HMMs according to the mechanical part. To know whether a mechanical part is abnormal or not, a test sequence of the considered time interval obtained by the process of Figure 10 is applied to the HMMs and then probabilities representing the degree of correspondence with each HMM are calculated. This is the evaluation problem of the HMM [15]. The probabilities are compared with each other and then the HMM having the high probability is selected as the model expressing the situation of the time interval. That is, if HMM related to an abnormal state that has a high probability, it means that the mechanical system is abnormal. 

### 4.2. Performance of the Proposed Algorithm

To evaluate the performance of the fault detection algorithm using HMM, all vibration signals are transformed into a sequence indicating operating state. A test sequence consists of 100 indicators and its duration is 100 s. Test sequences for abnormal state includes more than 15 indicators ‘C’ or ‘W’ and for normal state, lower than 5.

Table 5 shows the number of test sequences used for a performance check on each mechanical part. Sequences used in HMM training are not included in the test sequence. The number of test sequences for an abnormal state is less than for the normal state because there are few *caution* and *warning* states during wind turbine operation. For this, some metrics for measuring the performance used for the binary classification problem with imbalanced dataset are introduced as shown in Table 6. *Accuracy* is the number of correct predictions divided by the total number of test sequences. *TPrate* and *recall* are the number of correct abnormal predictions divided by the number of test sequences predicted as ‘abnormal’. *FPrate* is the number of wrong normal predictions divided by the number of test sequences predicted as ‘normal’. *Precision* is the number of correct abnormal predictions divided by the number of test sequences for ‘abnormal’. *F-measure* is defined as follows:

(4) $$F-measure=\frac{2\cdot Recall\cdot Precision}{Recall+Precision}.$$

All metrics except *FPrate* show high performance when they have high values. On the other hand, small value of *FPrate* means high performance [26,27]. 

In Table 6, judging from the *FPrate* and *precision*, all test sequences for abnormal state are perfectly indicated as ‘abnormal’. High *accuracy* (more than 0.96) means that prediction using the suggested HMMs has very low error. For the generator, however, *TPrate* is 0.573 because the number of test sequences of abnormal state of the generator is only 4.4% of the number of test sequences of its normal state. It is expected to improve as the number of test sequences of abnormal state increases. As a result, these measures mean that detection performance using the suggested HMMs is good. 

**Figure 12:**
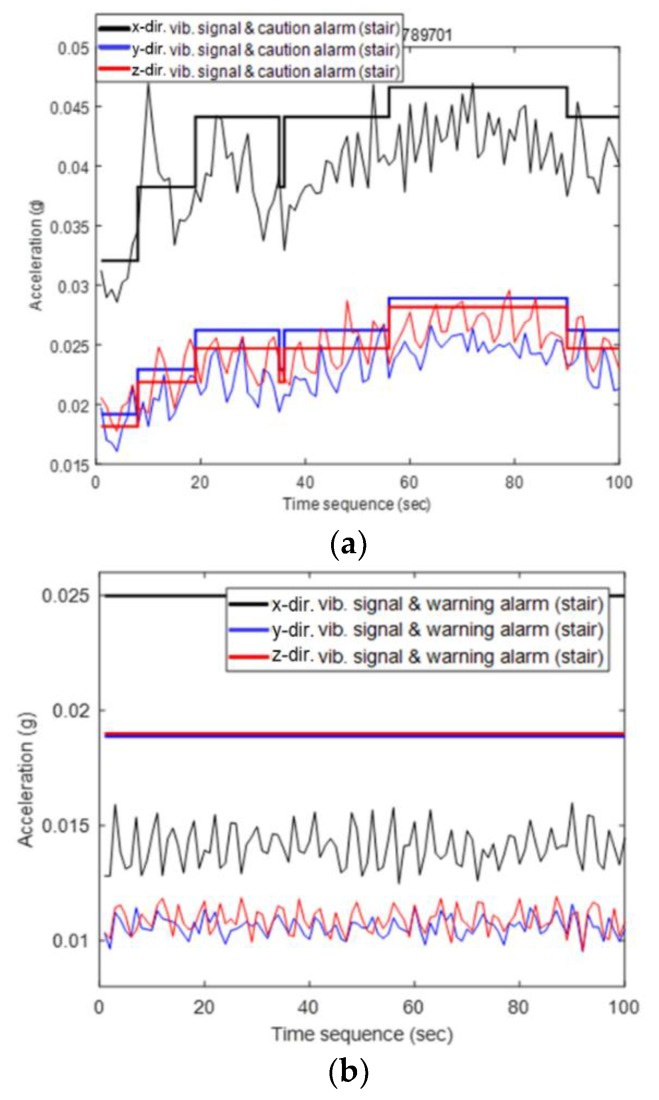
Vibrations of time duration judged as (**a**) abnormal and (**b**) normal by the proposed fault detection algorithm. The threshold level are also plotted which vary with rotational speed of the main shaft and with the vibrations are measured at the bearing of the main shaft.

The next step of the performance check is the fault detection process, wherein HMM is applied to the entire dataset and abnormal data are extracted. Figure 12 shows examples of data judged as abnormal or normal. 

This includes the vibration signals of x-, y-, and z- directions measured at the bearing of the main shaft and the thresholds related to a caution alarm. The threshold value changes as a function of the rpm of the main shaft, as mentioned in Section 3.2. Figure 12a shows an example of abnormal data. Some vibrations over the threshold value of caution appear in the x- and z- directions. That is the moment that the revolution of main shaft changes from 10 rpm up to 14 rpm and power is generated. In Figure 12b, which represents a normal condition, all vibrations are below the unchanged threshold because the rotational speed is constant at 8 rpm. Judging from the results, it can be concluded that it is possible to detect the time domain in which the abnormality occurs by applying the proposed algorithm which includes HMM.

## 5. Conclusions

This study proposed a fault detection algorithm using HMM to recognize whether mechanical parts of a wind turbine are behaving abnormally or not. A vibration signal was selected to determine the status of the wind turbine and acceleration signals were measured at the bearing of the main shaft, gearbox, and generator for more than 2 years. It was found that the distribution of the long-term vibrations could be approximated with the Weibull probability density function when the vibrations were classified by the rotational speed of main shaft. And then, the probability function was used to determine the threshold values indicating alarm levels. 

The input sequence for HMM was obtained by applying the threshold levels and the correlation between the vibration signals. Because HMM took into account the variation of the status during the a given time interval, it could overcome the disadvantage that the conventional methods exhibited, alarm errors due to one-off external impact signals due to either system starts or stops or unexpected electrical noise at a specific channel. As a result, it was found that the proposed HMM algorithm for fault detection achieved 96% *accuracy,* 0% *FPrate*, and 100% *precision* by analyzing the long-term vibration signals. 

In fact, it is not easy to obtain the vibration data that is directly related to the fault since there are very few actual fault events in the mechanical parts of the wind turbine. To overcome this, it will be necessary to improve the statistical reliability of the proposed HMM by adjusting the threshold level during continuous data acquisitions and re-training the transient probability distribution and the observation symbol probability distribution. In addition, as further works, the number of detectable states can be subdivided as using the advantage of HMM that can freely expand the number of classes and the performance needs to be compared with the results obtained from other classification methods. 

## Figures and Tables

**Table 1:** Vibration signals treated in this study. Here, RMS means the root mean square value of vibration signal below 1 kHz at an interval of 1 s and HFBP the root mean square value of vibration signal above 1 kHz. X, Y, and Z indicate the vibration directions: horizontal, vertical, and axial to the main shaft.

Mechanical Part		Acceleration Directions		
		X (Horizontal)	Y (Vertical)	Z (Axial)
*Main shaft bearing*		RMS	RMS	RMS
		-	HFBP	HFBP
*Gearbox*	*Low-speed part*	-	RMS	RMS
		-	HFBP	HFBP
	*High-speed part*	-	RMS	RMS
		-	HFBP	HFBP
*Generator*	*Input part*	-	RMS	RMS
		-	HFBP	HFBP
	*Output part*	-	RMS	RMS
		-	HFBP	HFBP

**Table 2:** Thresholds of vibration according to alarm level defined in this study. The *cdf* is the cumulative distribution function of Weibull distribution.

Alarm Level	Threshold, *Tr*	Alarm Range of Vibration, x
Normal	-	*cdf(x)* < 84.1%
Attention	*cdf(Tr)* = 84.1%	84.1% *≤* *cdf(x)* < 97.7%
Caution	*cdf(Tr)* = 97.7%	97.7% *≤* *cdf(x)* < 99.8%
Warning	*cdf(Tr)* = 99.8%	99.8% *≤* *cdf(x)*

**Table 3:** Number of training sequences for the HMMs related to mechanical parts.

Mechanical Part	For Abnormal	For Normal
*Bearing of main shaft*	2250	4500
*Gearbox*	910	4500
*Generator*	2250	4500

**Table 4:** Transient probability distribution and observation symbol probability distribution of the HMMs for each mechanical part of the wind turbine used in this study.

Mechanical Part	HMM	Transient Probability Distribution (2 × 2)		Observation Symbol Probability Distribution (2 × 4)			
*Bearing of main shaft*	For abnormal	0.945171	0.054829	0.678136	0.303485	0.018101	0.000278
		0.00676	0.99324	4.11×10^−8^	0.029147	0.361074	0.609779
	For normal	0.987934	0.012066	0.933989	0.065805	0.000203	2.27×10^−6^
		0.023135	0.976865	0.210395	0.697227	0.09153	0.000848
*Gearbox*	For abnormal	0.933872	0.066128	0.129456	0.851896	0.012909	0.005739
		0.02293	0.97707	6.66×10^−6^	0.003044	0.727025	0.269925
	For normal	0.954631	0.045369	0.960139	0.039777	7.67×10^−5^	7.92×10^−6^
		0.050568	0.949432	0.03402	0.931385	0.034524	7.11×10^−5^
*Generator*	For abnormal	0.926132	0.073868	0.264596	0.258718	0.457806	0.018881
		0.020584	0.979416	1.60×10^−41^	0.000315	0.142944	0.856741
	For normal	0.990231	0.009769	0.993997	0.005972	1.48×10^−5^	1.57×10^−5^
		0.044451	0.955549	0.094724	0.809085	0.095486	0.000705

**Table 5:** Number of test sequences for the HMMs related to mechanical parts.

Mechanical Part	For Abnormal	For Normal	Total # of Test Sequences
*Bearing of main shaft*	1628	5843	7471
*Gearbox*	364	6406	6770
*Generator*	294	6622	6916

**Table 6:** Performance measures of the suggested HMM by introducing metrics used for imbalanced binary classification.

Mechanical Part	*Accuracy*	*TPrate (Recall)*	*FPrate*	*Precision*	*F-Measure*
*Bearing of main shaft*	0.961	0.848	0.000	1.000	0.918
*Gearbox*	0.986	0.791	0.000	1.000	0.884
*Generator*	0.968	0.573	0.000	1.000	0.729
